# Northern Ohio Dental Association

**Published:** 1871-07

**Authors:** H. H. Newton


### Proceedings of Societies.

### NORTHERN OHIO DENTAL ASSOCIATION.

PUT-IN-BAY, May 2nd, 1871, 10 o'clock, A. M.

The association met according to the requirements of the Constitution.

Dr. J. E. Robinson, President, in the chair.

Meeting called to order. Minutes of previous meeting read and approved.

Members present: J. E. Robinson, B. F. Robinson, L. Buffett, C. Buffett, H. H. Newton, Cleveland; E. J. Waye, Sandusky; J. F. Siddall, Oberlin; C. B. Knowlton, Elyria; D. R. Jennings, Ravenna; Prof. Taft, Cincinnati, and several visitors.

The report of the Treasurer was referred to an Auditing Committee. Dr. Siddall appointed as Auditor.

Chair appointed as Examining Committee, Drs. L. Buffett, D. R. Jennings and B. F. Robinson.

Dr. Siddall appointed as Treasurer, *pro tem.*

Adjourned to 2 o'clock P. M.

### AFTERNOON SESSION.

Meeting called to order by the President.

The Examining Committee presented and recommended the following persons for membership, viz: Dr. J. Stephan, Cleveland; Dr. R. R. Peebles, Cleveland; Dr. H. M. Ried, Sandusky; Dr. A F. Price, Fremont.

All of whom were duly elected by ballot.

DISCUSSIONS.

*First—The benefits of a thorough Dental Education.*

Dr. L. Buffett: In discussing a subject of so vital importance, the first inquiry should be, What constitutes a thorough Dental education? First, we should have a general knowledge of anatomy, physiology, medicine and surgery, that we may give our patients the benefit of our best skill. We should also have a knowledge of metallurgy and chemistry. Beyond this a dentist should be a good mechanic, and his mechanical powers should be so trained and educated, that his manipulative ability may be brought to great nicety and perfection.

Dr. Siddall: Regards our position before the public, is such that we should secure to ourselves a great degree of excellence, in order that the public may not be disappointed in us. We should rank with other professions.

Dr. J. E. Robinson: Feels the need of a more thorough dental education. The subject is of such importance that we should more thoroughly understand our calling, in order that we may give the people our best services, which they as recipients demand.

Dr. B. F. Robinson: Can not speak so definitely of the benefit of a thorough dental education, as of the want. Had been in the profession many years; commenced when the opportunities for acquiring a dental education were not as now, but still saw the necessities of it more than ever before.

Dr. Knowlton: Anatomy and physiology should be thoroughly understood by every dentist, if he wishes to confer the best benefit upon his patients. The people demand at his hand a thorough knowledge of his profession.

Dr. C. Buffett: The moral worth and honesty of a dentist is of the greatest importance. If a man is conscientious he will do good work, and not pretend he can do a thing unless he can do it thoroughly.

Dr. Peebles: The best time to obtain a dental education is before we have much practice. He was trying to profit by the experience of others, as well as by all cases that are presented in his daily practice, feeling deeply that our patients require at our hands our best knowledge and ability.

Dr. Ried: Our greatest need is a more thorough knowledge of our text books, which pertain to the various branches.

Dr. Waye: A man of the present day does not know the advantages he has over one of twenty-five years ago. The student of to-day has a fine chance for a thorough college course, which is of incalculable value to him in future practice.

*Second—The proper Treatmrnt of Children's Teeth,*

Dr. L. Buffett: The profession are divided on the subject of the eruption of children's teeth. The cause of the suffering of children with their teeth, is the want of a proper nutrition. We, as dentists, have much to investigate in regard to the management of children's teeth. We should educate the people, especially mothers, to the idea that their children should have the kind of food that contain a proper amount of *lime*, to make strong and healthy bone. They should learn that candies, sweetmeats, etc., can not be eaten without harm. It belongs to the dentist to look into this subject and ascertain the wants of the child. On the subject of filling children's teeth, would use a non-conductor, (Hill's Stopping.) and if it should wear out, refill.

Dr. Waye: Has many doubts what to do in severe cases of inflammation. Has made attempts to fill the roots of children's teeth, but has failed. Is of the opinion that the temporary teeth should be filled and retained as long as possible.

Dr. Peebles: On the subject of severe inflammation, would use the lance, had found it an instantaneous relief. It was his opinion that it was better to lose a permanent tooth, than a temporary one prematurely.

Dr. J. E. Robinson: His method of treatment for exposed pulps in children's teeth, is to apply arsenic and creosote in very minute doses, then remove the dead nerve and fill, and retain them as long as possible. Considers the retention of the temporary teeth as long as nature requires, as of the utmost importance. They should never be extracted unless in cases of extreme suffering.

Dr. Taft: On exposure of dental pulp, would paliate rather than destroy, but would not use arsenic, but alum in sulph. ether with cotton, (a fine treatment in some cases,) then fills the cavities with Hill's Stopping; sometimes covers the pulp with collodion, and then fills with os-artificial. When the pulp is found dead, removes the debris. Very great care should be maintained to preserve the deciduous teeth. The roots should not be filled with any hard material. The retention of the ulcerated teeth depends upon circumstances. If the inflammation can not be subdued, extract them. There are many irregularities made by the premature extraction of the deciduous teeth, which causes a contraction, or prevents the proper expansion of the jaw to accommodate the permanent teeth. One very important thing parents should understand is, the proper use of these teeth in masticating food, and keeping them clean. Hard food is better than soft, as it gives the teeth something to do. Would fill the teeth with whatever material would preserve them best. (Dr. Taft in answer to a question.) Could see no evil results in lancing the gum in severe inflammation, but much good.

*Third—Diseases of the Gums and Alveolar Processes.*

Dr. Taft: When the functions of the mucous membrane upon the gums have so changed as to act injuriously upon the dentine at the necks of teeth, great sensitiveness, occurs, and ultimately decay. The cause of such a condition is a change in the mucous follicles of the gums.

Dr. L. Buffett: There must be an irritated condition of the gums. Is it a local trouble, or is it some difficulty of the system, which produces an acid condition and causes decay?

Dr. Taft: The irritation of the mouth is a very serious difficulty in the wearing of dental plates, especially rubber and pyroxilyn, in many cases effecting the system injuriously. The majority, however, can wear gold plates without any serious objection.

Adjourned until to-morrow, 8½ o'clock A. M.

SECOND DAY.

The convention met according to adjournment.

The Auditor's report was received and adopted.

Dr. J. E. Robinson remarked that he had at first found considerable difficulty in the use of the rubber dam. It producing severe inflammation about the necks of the teeth, in forcing it down to its place, but had now in a manner overcome it.

Dr. Taft: Used the rubber dam, but had no cases of severe inflammation from its use. He could perceive how the rubber and ligature might impinge upon the neck of the tooth, so hard as to induce inflammatory action. Would use iodine after the operation as a remedy to counteract this inflammation.

Dr. L. Buffett: Would like some light upon the subject of necrosis before and after extracting. Had one case of two years standing of the inferior maxillary. Cut away the loose pieces which resulted in a cure.

Dr. Price: Related an interesting case of ulceration and necrosis of the inferior maxillary, which by removing the tooth and probing the parts, the necrosed bone is in the process of being exfoliated. The pain has ceased and a cure is indicated. The patient has a scrofulous indication.

Dr. Taft: In cases of necrosis, a good remedy is sulphuric acid, applied once in two days, directly to the bone, using care in its application not to touch the soft parts with the acid. Has used it in several cases with very good results. Was first suggested to him by Dr. Atkinson.

Dr. Jennings: In cases of a diseased condition of the margins of the gums, sulphate of zinc is a very good remedy applied carefully to the parts effected.

10 o'clock, A M.

The time had now arrived for the election of officers for the ensuing year, which resulted as follows:

*President*—E. J. Waye.

*Vice President*—J. F. Siddall.

*Recording Secretary*—H. H. Newton.

*Corresponding Secretary*—J. E. Robinson.

*Treasurer*—C. Buffett.

*Examining Committee*—B. F. Robinson, D. R. Jennings, W. P. Horton.

*Delegates to American Dental Association*—L. Buffett, E. J. Waye, D. R. Jennings, B. F. Robinson, R. R. Peebles, C. Buffett, C. R. Butler.

Dr. E J. Waye, was conducted to the chair, and thanked the association for the honor conferred upon him in his election as their presiding officer, and would endeavor to discharge its duties for the welfare and progress of the dental profession.

Dr. J. E. Robinson, (the retiring President) then gave a very interesting address upon the progress of dental science, and importance to humanity, and wishing us God speed in all of our laudable undertakings.

On motion, the Committee on Dental Ethics was dissolved.

Resolution offered by Dr. B. F. Robinson, That article 9 of the Constitution, be amended by striking out the word *first* Tuesday of May, and inserting the *last* Tuesday of May.' Carried.

On motion, it was resolved to meet on the last Tuesday of May, 1872, at Put-in-Bay.

*Fourth—Heavy Foils and Heavy Mallets.*

Dr. J E. Robinson: Uses a lead mallet of 3½ ounces, likes it better than any he has used. He is of the opinion that heavy foil can be more closely packed into a tooth than light foil.

Dr. L. Buffett: Has used both heavy and light foil; was of the opinion that the light foil was more safe in frail teeth, and more readily adapted to the walls than the heavy numbers. We should always use our best judgment, and select that foil which is best adapted to the case in hand.

Dr. Jennings: Likes the heavy foils, 30 and 60 in small cavities in front teeth.

Dr. Newton: Has used Nos. 4 and 6 mostly, and his experience is, that they are more readily adapted to the walls of small cavities, than the heavier numbers. Has used Nos. 20 and 30 to some extent on the surface of large fillings with satisfactory results. Uses a 4 ounce iron mallet, and prefers it to any other he has used.

Dr. Taft: Let every dentist use his best judgment in operating, so he may accomplish the best results to himself and patients. Has used many kinds of mallets, and finds that different patients like different mallets. In the use of the mallet, we should strike squarely upon the end. It is well for us to study the philosophy of the subject. The use of heavy foil in building up and overlapping the thin edge of a tooth, is of great value, and should always be done so as to perfectly protect and prevent the walls of the tooth from breaking down in the process of mastication. There are many cases that fail entirely from this cause alone. In filling small cavities he uses magnifying eye glasses, and finds them a very great advantage in preparing cavities, and recommends their use, that we may better perform our work. No injury can result to the eyes from their use.

Adjourned, to meet at Put-in-Bay, on the last Tuesday of May, 1872.

H. H. Newton, *Rec. Sec.*

Editor Dental Register: Noticing in the late proceedings of the Ohio Dental Society, that the influence of malaria was referred to, suggested to the writer that it might prove acceptable to offer a few observations made while in a miasmatic region. During five years practice as dentist in that highly malarious district, comprising in part a portion of the lower Wabash valley; experience led me to believe that tumors seen on the gums of patients, were of a benign rather than of a malignant nature. I can recall to mind but two cases during that time, where the tumor was marked by unusual size, rapidity of growth, or from the presence of which, the general health was seemingly disturbed. One vexatious trouble met with was, a lengthening and thickening of the gums after extraction of teeth, at such time the edges presenting a plump and glazed appearance. There being no complaint of derangement of the functions, local treatment was recommended which was followed in a majority of cases, by a partial absorption of the substance only. A neighboring dentist, who had himself experienced like trouble in his practice, attributed the cause to malaria; he recommending as treatment, purgatives, followed by bark tonics, "in some cases the result was satisfactory." I thought then, and believe now, that the true cause was traceable to the depraved state of the system. In the more unfavorable parts of the above locality, a majority of the inhabitants seem more or less tainted with scrofula—in connection with which we find quinsy, chronic enlargement of the cervical glands, and the appearance of simple white ulcers on the tongue. Patent nostrums, traveling quacks, and old women's cures are patronized extensively, while the advice and treatment of the physician is too often ignored. In conclusion, I would say, that what appeared to me strange, was that in a locality where above all others, excitent cause would seem to exist, and where the dentist would naturally look for tumors to assume a malignant form, we should find instead, those of a benign nature. J.

